# Mucosal Type of Chronic Suppurative Otitis Media and the Long-Term Impact on Hearing Loss

**DOI:** 10.7759/cureus.10176

**Published:** 2020-09-01

**Authors:** Muhammad Salar-e-Azam Rajput, Muhammad Shaheryar Ahmed Rajput, Asif Ali Arain, Syed S Zaidi, Ahmad Hatem, Saeed Akram

**Affiliations:** 1 Otolaryngology and Head & Neck Surgery, Liaquat University Hospital, Hyderabad, PAK; 2 Otolaryngology and Head & Neck Surgery, Liaquat University of Medical and Health Sciences, Jamshoro, PAK; 3 Otolaryngology and Head & Neck Surgery, King Faisal Specialist Hospital and Research Centre, Riyadh, SAU; 4 Otolaryngology and Head & Neck Surgery, Aga Khan University Hospital, Karachi, PAK; 5 Otolaryngology and Head & Neck Surgery, The Indus Hospital, Karachi, PAK; 6 Internal Medicine, King Faisal Specialist Hospital and Research Centre, Riyadh, SAU

**Keywords:** hearing loss, chronic otitis media, mucosal disease, csom, snhl, mixed hearing loss

## Abstract

Introduction

Worldwide the chronic suppurative otitis media (CSOM) is one of the most common infectious diseases in childhood and is a common cause of impaired hearing. The disease remains a challenging entity for the healthcare system of resource-limited nations despite the advances in modern medicine. The nature of hearing loss in CSOM is mainly conductive, the sensorineural hearing loss (SNHL) is also reported in such patients. The purpose of the study was to identify SNHL in patients with the mucosal type of CSOM and to find the impact of long-term discharging ears on bone conduction (BC) thresholds.

Methods

Patients with a diagnosis of the mucosal type of CSOM were identified from the record of ENT, Head and Neck Surgery clinic between January 2019 and January 2020. The patients were divided into three groups based on the duration of the disease: groups I, II, and III for 1-5 years, 5-10 years, and 10-15 years, respectively. Pure tone audiogram was reviewed, and data of BC was recorded for 500, 1000, and 2000 Hz. The descriptive frequency was calculated for SNHL in each group and group I was compared with other groups using a chi-square test. The mean BC threshold of group I was compared with other groups using a t-test. SPSS version 26 (IBM Corp., Armonk, New York) was used for statistical analysis.

Results

A total of 154 patients were included in the study. There were 73 males and 81 females. The mean age was 26 years. The minimum age was 12 years and the maximum age was 58 years. Active ear discharge was the presenting complaint in 84 patients. The right ear was involved in 88 patients, and the left ear was involved in 66 patients. SNHL was present in 30 out of 154 patients, i.e., 19.5%. The number of patients in each of the groups I, II, and III was 95, 28, and 31, respectively. The group I was compared with group II using the chi-square test, the p-value was found not significant, i.e., >0.05. The group I was then compared with group III using the same statistical test, and the p-value was found significant, i.e., <0.05.

The mean BC threshold for an average of three speech frequencies for each of the three groups was 16.9, 18.7, and 22.9, respectively. The mean BC threshold of group I was compared with that of group II using a t-test, and the p-value was found not significant, i.e., >0.05. The mean BC threshold of group I was then compared with that of group III using a t-test, and the p-value was found significant, i.e., <0.05.

Conclusions

The findings of our study reproduce the presence of SNHL in a sizable proportion of the patients with a mucosal type of CSOM. Furthermore, the elevation of the BC threshold also appears statistically significant on analysis in association with the protracted duration of CSOM, highlighting the adverse impact of delaying the surgical repair. However, the clinical importance remains unclear because the maximum losses in the BC threshold seen in the patients are not severe enough to necessarily make them hard of hearing. Nevertheless, these statistically significant results influence clinical thought process and measures for an early remedy, including surgery, and need to be considered in time to prevent progressively worsening hearing loss in such cases.

## Introduction

Chronic suppurative otitis media (CSOM) is an inflammation of the middle ear associated with infection and characterized by a persistent otorrhoea from a perforated tympanic membrane over a month [1-3]. Two types of CSOM are recognized, a mucosal disease (safe type) and an epithelial disease (unsafe type) [3]. Worldwide the CSOM is one of the most common infectious diseases in childhood and is a common cause of impaired hearing, in particular, in resource-limited parts of the world [4,5].

Multiple episodes of acute otitis media often precede CSOM, which develops as a consequence when the natural repair mechanism of the tympanic membrane fails to completely heal the perforation. Once the CSOM is developed in early childhood it has the potential to spill over into adulthood and becomes the cause of recurring episodes of discharging ears unless the drum is repaired surgically. Therefore in adult patients, the disease usually has a long history spanning over years or even multiple decades [6,7].

The disease remains a challenging entity for the healthcare system of developing nations due to the enormous economic burden with a prevalence of 72 per thousand population [8]. Despite the many advances in health care and surgical practices globally, the impact of CSOM is overwhelming and often more noticeable in the developing countries [9].

The contributing factors to the high proportions of CSOM in these regions are over-crowding, malnutrition, poor hygiene, colonization with potentially pathogenic microorganisms in the nose and nasopharynx, and lack of easy access to standard healthcare facilities that are already very limited [10]. *Pseudomonas aeruginosa*, *Staphylococcus aureus*, *Klebsiella pneumonia*, *Escherichia coli*, and anaerobes are common causative bacteria reported in most cases of chronic otitis media [11].

However the nature of hearing loss in CSOM is often conductive due to damage to tympanic membrane or ossicles, the sensorineural hearing loss (SNHL) is also reported in such patients [12,13]. The purpose of this study was to identify SNHL in patients with the mucosal type of CSOM and to find the impact of duration of the discharging ears on bone conduction (BC) thresholds.

## Materials and methods

Patients with a diagnosis of the mucosal type of CSOM were identified from the record of ENT, Head and Neck Surgery clinic from January 2019 to January 2020. The criteria for inclusion and exclusion are shown in Table 1.

**Table 1:** Inclusion and exclusion criteria PTA: pure tone audiogram

Inclusion Criteria	Exclusion Criteria
Unilateral disease	History of head injury
Normal hearing in the opposite ear on PTA	History of meningitis
No previous otological or brain surgery	Incomplete record or missing PTA

A total of 154 patients were included in the study. Based on the duration of the disease, patients were divided into three groups: groups I, II, and III for 1-5 years, 5-10 years, and 10-15 years, respectively. Pure tone audiogram (PTA), a routine investigation for hearing assessment, was reviewed, and data of BC for three speech frequencies 500, 1000, and 2000 Hz were recorded for statistical analysis in SPSS version 26 (IBM Corp., Armonk, New York). Average was calculated for three speech frequencies, and threshold > 25 decibels (dB) was taken as a cutoff for the presence of SNHL. The descriptive frequency was calculated to identify the number of patients with SNHL in each group, and group I was compared with other groups using a chi-square test. The mean was computed for BC thresholds in each group, and group I was compared with other groups using a t-test to find the long-term impact of CSOM (mucosal type) on BC.

## Results

Out of 154 patients, there were 73 males and 81 females. The mean age was 26 years. The minimum age was 12 years, and the maximum age was 58 years. Active ear discharge was the presenting complaint in 84 patients. The right ear was involved in 88 patients, and the left ear was involved in 66 patients. SNHL was present in 30 out of 154 patients, i.e., 19.5%. These variables are shown in Table 2.

**Table 2:** Age, gender, and clinical variables of the patients SNHL: sensorineural hearing loss

Variables		Results
Gender	Male	73
	Female	81
Age	Mean	26 years
	Range	12-58 years
Ear discharge	Yes	84
	No	70
Laterality	Right ear	88
	Left ear	66
SNHL	Yes	30
	No	124

The number of patients in each of the groups I, II, and III was 95, 28, and 31, respectively. The group I was compared with group II using the chi-square test; the p-value was found not significant, i.e., >0.05, as shown in Table 3.

**Table 3:** SNHL and comparison between groups I and II SNHL: sensorineural hearing loss

		Disease Duration		Chi-Square Test
		Group I (1-5 years)	Group II (5-10 years)	p-value
SNHL	Yes	14	06	0.399
	No	81	22	
Total		95	28	

The group I was then compared with group III using the same statistical test, and the p-value was found significant, i.e., <0.05 as shown in Table 4.

**Table 4:** SNHL and comparison between groups I and III SNHL: sensorineural hearing loss

		Disease Duration		Chi-Square Test
		Group I (1-5 years)	Group III (10-15 years)	p-value
SNHL	Yes	14	10	0.031
	No	81	21	
Total		95	31	

The mean BC threshold for an average of three speech frequencies for each of the three groups was 16.9, 18.7, and 22.9, respectively. The mean BC threshold of group I was compared with that of group II using a t-test, and the p-value was found not significant, i.e., >0.05 as shown in Table 5.

**Table 5:** Mean bone conduction threshold and comparison between groups I and II N: number of patients in each group

Disease Duration	N	Mean	Standard Deviation	t-test
Group I (1-5 years)	95	16.9	7.13	p-value 0.23
Group II (5-10 years)	28	18.7	6.02	

The mean BC threshold of group I was then compared with that of group III using a t-test, and the p-value was found significant, i.e., <0.05 as shown in Table 6.

**Table 6:** Mean bone conduction threshold and comparison between groups I and III N: number of patients in each group

Disease Duration	N	Mean	Standard Deviation	t-test
Group I (1-5 years)	95	16.9	7.13	p-value < 0.001
Group III (10-15 years)	31	22.9	8.12	

## Discussion

Despite the several reports of an association between CSOM and SNHL, the consensus is still lacking about its importance. The study published in 1984 reported SNHL in the study subjects with chronic otitis media and suggested passage of inflammatory agents through the round window as a possible mechanism of cochlear damage resulting in SNHL. Additionally, it was considered the anatomical position and characteristic of the round window encourage this passage. There were two groups of patients based on the severity of hearing loss. The authors found 43% of ears with unilateral disease and 42% of ears with bilateral disease showed losses 15 dB or greater; group 1. In group 2, 16% of ears with unilateral disease and 17% of ears with bilateral disease revealed losses 30 dB or greater. The study did not consider the effect of the duration of the disease and the use of ototoxic ear drops [14].

In 1998, El-Sayed reported the higher BC thresholds over a number of frequencies by 9 to 14 dB. Twelve percent of the patients had a difference of 20 dB or more when compared to the opposite normal ears, and 39% of the patient had a difference of 10 dB or more [15]. Yoshida et al. in 2009 reported the percentage of patients with SNHL was likely to increase with age [16].

The conductive hearing loss which is constantly associated with CSOM is responsive to surgical treatment. On the other hand, the SNHL that follows a few years later is the result of permanent damage to the cochlea. If the treatment is delayed for years or decades the possibility of the need for a hearing aid, even after the surgical correction of the conductive defect, increases with protracting duration of the disease and recurrent infections [17].

Furthermore, a majority of adults do not want to use hearing aids (HA). A number of explanations were given, including the hearing aid value, fitting and maintenance issues, patient’s attitude, economical factors, psychosocial reasons, appearance, etc. McCormack and Fortnum conducted a study on reasons why people fitted with HA do not wear them. The study concluded that the most important issues were about the value of a hearing aid. Often a hearing aid does not provide enough benefit, or wearing a HA is not comfortable [18].

Similar results were also reported by several other studies [19,20]. These findings support the hypothesis that put forward inflammation in the middle ear may alter the permeability of round window membrane (RWM); therefore the bacteria and their remnants, for example, endotoxins, may perhaps pass through and adversely affect the inner ear functions, especially the high-frequency region which is anatomically located next to the round window [21,22].

The RWM is the only soft tissue and very thin structure that separates the inner ear from the middle ear. As a consequence, the assumption is made that inner ear damage happens as the effects of changes in the permeability of the RWM. To apprehend better the pathogenesis of inner ear impairment caused by middle ear inflammation or infection, the RWM structure was best investigated in rat models [23]. Several studies reported inflammatory cell infiltration and penetration of bacteria into the RWM and neighboring scala tympani [24,25].

## Conclusions

The number of patients revealed the presence of SNHL is quite significant. The statistical analysis further highlights the worse impact on BC thresholds due to the protracted course of the disease, which is inevitably associated with recurrent middle ear infections. The measures for early surgical treatment are recommended to avoid progressive hearing loss in these cases.
